# Leveraging the interpersonal context of child development to promote family resilience: A universal prevention approach from preconception through early childhood

**DOI:** 10.1016/j.mhp.2024.200331

**Published:** 2024-02-15

**Authors:** Jennifer A. Somers, Laura K. Winstone-Weide, Gabrielle R. Rinne, Sarah G. Curci, Margot E. Barclay

**Affiliations:** a Department of Psychology, University of California, Los Angeles, CA, USA; b Department of Neurology, University of Texas Austin, Dell Medical School, USA; c Department of Psychology, Arizona State University, Tempe, AZ, USA

**Keywords:** Universal prevention, Relational health, Family resilience

## Abstract

Significant mental health problems affect one in five youth in the United States; in tandem with the child mental health epidemic, parents in the United States report high and rising rates of burnout and mental health challenges of their own. Multiple well-established theoretical perspectives demonstrate the high degree of interdependence between children’s and their parents’ mental health, including intergenerational transmission, prenatal programming, attachment, and temperament and self-regulation theories. Drawing on these perspectives, we argue that a *universal prevention* approach that centers the development of psychopathology within the context of the parent-child dyad can promote resilience and arrest emerging mental health problems for children and their parents, during sensitive developmental windows (e.g., preconception through early childhood). Derived from this integrated theoretical framework, we review empirical support for the following targets to promote family resilience: screening for current and historical parent risk factors and resilience resources; strengthening healthy, reciprocal social ties; and supporting youth socioemotional skill acquisition. Our review of the literature highlights how improvements in these areas can have cascading benefits across development, for both parents and their children, as well as for future generations. We conclude with actionable, empirically-supported recommendations that can have profound impacts on these targets through changes in federal and state policies, community healthcare settings, and early childhood education and care programs. To achieve enduring, multigenerational impacts, societal and community-level policies, programs, and practices must interweave efforts to support child mental health with efforts to promote parent adjustment and wellbeing.

Large-scale epidemiological research provides compelling evidence that most mental illnesses emerge during childhood or adolescence (Solmi et al., 2022), warranting concerns about the current and future burden associated with increasing rates of childhood mental health problems in the United States (Lebrun-Harris et al., 2022). In tandem with the child mental health crisis (Office of the U.S. Surgeon General, 2021), adult caregivers (hereafter referred to as ‘parents’) report high rates of parenting stress and mental health challenges of their own (Lebrun-Harris et al., 2022). Critically, parent and child mental health problems frequently co-occur (Patrick et al., 2020) such that one family member’s problems can also spillover and increase risk for other family members (e.g., Curci et al., 2023; Roubinov et al., 2021). Due to their high degree of interdependence, it is imperative to *prevent* mental health problems in *both* children and their parents,^2^ especially as many children and parents either cannot obtain or do not respond to existing mental health services (Merikangas, Nakamura & Kessler, 2009; Whitney & Peterson, 2019). Prevention efforts that leverage the interpersonal context of child psychopathology, particularly during sensitive periods for biological embedding of close relationships (e.g., 0–5 years; Gee & Cohodes, 2021; Luby, 2022), hold potential cascading benefits for not only this generation’s longevity and productivity but also for those of their parents and future generations (Masten et al., 1999; McLuckie et al., 2019; Roubinov et al., 2021). In this paper, we contend that a *universal prevention* approach that centers the development of psychopathology within the context of the parent-child dyad can promote resilience to mental health problems, across diagnostic taxa, for young children and their parents. We offer an empirically-supported framework for specific targets to support relational health early in child development and prevent mental health problems in parents and children, followed by specific policy recommendations in the United States.

## 1. A relational health approach to mental health and prevention

At the core of the relational health framework is the potential for close relationships to buffer against the physiological toll of adversity exposure and scaffold the acquisition of resilience resources, such as socioemotional skills (Garner & Yogman, 2021). Extending this top-down approach whereby close relationships with parents can “buffer” against stress (Gunnar & Hostinar, 2015; Vila, 2021), transactional models of development (e.g., Sameroff, 1983, 2009) highlight the *interconnectedness* between children and their parents. From this transactional perspective, child developmental outcomes are neither the outcome of individual nor experiential factors alone, but rather are the product of transactions within parent-child relationships (Sameroff 1983, 2009). Under this umbrella of *relational health* and *transactional models of development*, our approach integrates multiple well-established theoretical perspectives, including intergenerational transmission, attachment, and temperament and self-regulation theories, that underscore the interdependence between child and parent mental health.

The origins of psychopathology extend well before birth (Monk, Lugo-Candelas & Trumpff, 2019), and involve biobehavioral processes where parents influence *and* are influenced by their children across development (Roubinov et al., 2021). It is well-established that the psychological effects of parental trauma and mental health problems increase risk for offspring mood and behavior problems, a process referred to as “intergenerational transmission” (Bowers & Yehuda, 2016; Goodman et al., 2011; Masten & Cicchetti, 2016). Even parental distress prior to conception can bear negative consequences for fetal and infant stress physiology and neurodevelopment (Keenan et al., 2018; Moog et al., 2016; Swales et al., 2018; van den Bergh, 2020; Yehuda et al., 2009; Yehuda & Lehrner, 2018; Yehuda & Meaney, 2018). Similarly, parents’ childhood experiences shape their mental schema of how individuals learn, view, and interact with close others (Main et al., 1985; Raby & Dozier, 2019) which in turn predict their responsiveness, sensitivity, and support of their own children’s autonomy (Jones et al., 2015; Koehn & Kerns, 2018). Parents model and scaffold children’s acquisition of socioemotional skills and regulatory strategies for successful self-regulation (Eisenberg et al., 2005) as well as potent resilience resources (Holodynski, 2013), such as positive regulatory behaviors (e.g., positive appraisal style) and executive functioning (Fay-Stammbach, Hawes & Meredith, 2014).

Though researchers have often adopted a parent-driven model of child development, the development of children’s regulatory systems involves mutually reciprocal, synchronous exchanges between parents and their children. This dyadic biobehavioral synchrony - where parent and child each respond to the other’s changing emotional and physiological signals (Feldman, 2017) – and coregulatory interactions lay the foundation for attachment (Feldman, 2012) and shape the development of children’s biobehavioral regulatory systems and volitional self-regulation (Calkins & Marcovitch, 2010; Feldman, 2007). Yet both parental and child risk factors can compromise coregulatory dyadic functioning. Stressed mothers (e.g., depressed mothers and those exposed to early life adversity) may be less responsive to child cues and provide less adaptive models of co-regulation and self-regulation (Bernard-Bonnin, 2004; Goodman et al., 2011; Roubinov et al., 2021). Likewise, stress-exposed children may exhibit elevated negative emotional volatility (Somers & Luecken, 2022) and temperamental negative emotionality and learn maladaptive strategies for self-regulation (Bernard-Bonnin, 2004), which may evoke ineffective parenting behaviors, compromise dyadic functioning, and place both parent and child at increased risk for developing subsequent mental health difficulties (Belsky, 1984; Bornstein, 2019; Luecken et al., 2019; Troutman et al., 2012; Winstone et al., 2021). Taken together, promoting relational health and preventing mental health problems in young children and their parents requires intervening on the transacting relations between parent mental health that begin before birth and span development (Roubinov et al., 2021).

## 2. Relational health: targets for prevention

Derived from our transactional relational health framework, we provide empirical support for the following targets from preconception to age five that promote family resilience and prevent mental illness: (1) screening for current and historical parent risk and resilience factors; (2) strengthening healthy, reciprocal social ties; and (3) supporting youth socioemotional skill acquisition. Table 1 summarizes the strength of the evidence linking these recommended targets to parent and child mental health outcomes, and offers empirically-supported strategies for affecting change in these targets. Attention to the dyadic context of child psychopathology not only holds potential to prevent disorder transdiagnostically, but also to improve the lives of multiple generations.

### 2.1. Screening for historical and current parent risk factors and resilience resources

#### 2.1.1. Screening for risk

Across the prenatal, pregnancy, and postpartum (i.e., perinatal) periods, mothers undergo a host of biological, psychological, and social changes that confer increased vulnerability to stress and mental health problems; the U.S. Preventive Services Task Force recommends screening for depression and anxiety for pregnant women and women of reproductive age (O’Connor et al., 2023). Not only is postpartum depression associated with marital discord, family dysfunction, increased substance use, and higher healthcare costs, but it also poses a risk to dyadic functioning and children’s cognitive development, socioemotional functioning, and behavior (Earls, 2010; Essex et al., 2002). Either directly or indirectly via its effects on perinatal distress, the biological and behavioral impacts of adverse childhood experiences (ACEs) may also be particularly salient during the transition to new parenthood (Hays-Grudo et al., 2021; Roubinov et al., 2021).

#### 2.1.2. Screening for resilience

Whereas extensive research has emphasized identifying risk (especially elevated perinatal depression and anxiety symptoms) for poor maternal and infant health, more recent work highlights the importance of identifying salutary influences (Davis & Narayan, 2020), which may include benevolent experiences in mothers’ own childhoods (benevolent childhood experiences; BCEs; Davis & Narayan, 2020) and current resilience resources (protective and compensatory experiences; PACEs; Hays-Grudo et al., 2021), that attenuate risk and promote wellbeing. Higher levels of BCEs (e.g., supportive relationships, a favorable sense of self, and a predictable quality of life) predict fewer mental health problems and decreased exposure to stress in adulthood (Narayan et al., 2018, 2020), even after accounting for ACEs (Merrick & Narayan, 2020). Current PACEs that may interrupt negative developmental cascades (Hays-Grudo et al., 2021) consist of relationship factors (e.g., caregiver unconditional love, social group membership, having a best friend, community volunteering, mentorship) and environmental resource factors (e.g., home safety/cleanliness, pursuing a hobby, quality of education, family routines, and physical activity).

### 2.2. Strengthening healthy, reciprocal social ties

#### 2.2.1. Partner support

Beginning in the perinatal period, support from romantic and co-parenting partners and extended family members may play unique roles in promoting parental and child wellbeing (Coburn et al., 2016; Smith & Howard, 2008; Sufredini et al., 2022). Importantly, although parents’ experiences with their caregivers and adults in their own childhood may impact attachment schema and postpartum adjustment (Narayan et al., 2017, 2019), later attachment experiences with romantic partners may alter attachment status set forth in infancy, offering opportunities to transform insecure attachment to a more secure style (Booth-LaForce et al., 2014; Taylor et al., 2015; Thompson, 2000; Weinfield, Sroufe & Egeland., 2000). Support from co-parenting partners, including engagement in childcare, has the potential to promote positive parent and child functioning in the first few years of life (Coburn et al., 2016; Roubinov et al., 2021) and buffer the effects of maternal early life adversity and current contextual stressors on post-partum maternal mental health, parenting, and infant health and childhood behavior problems (Atzl et al., 2019; Luecken et al., 2013; Sarkadi et al., 2008; Stapleton et al., 2012). Such effects may operate through benefits for children’s emotion regulation skill acquisition: notably, these influences are likely bidirectional, as well-coordinated co-parenting enhances child emotion regulation and is also facilitated when parents are rearing a well-regulated child (Paley & Hajal, 2022).

#### 2.2.2. General social support

More generally, there is well-replicated evidence that receipt of social support from friends and family in the first year postpartum is associated with more sensitive maternal caregiving, a well-established predictor of child emotion regulation skills (Paley & Hajal, 2022), and less maternal stress, depression, anxiety, and self-harm (Bedaso et al., 2021; Ni & Siew Lien, 2024; Razurel et al., 2013). Frequent and high-quality social support for parents during the perinatal period may promote adaptive caregiver-child dyadic interactions and subsequent infant development indirectly via reduced postpartum mood and anxiety disorder symptoms (Feinberg et al., 2022; Luecken et al., 2015; Racine et al., 2020; Takacs et al., 2021; Thomas et al., 2017). Informal support, such as support from partner, family, and community members, has been found to be preferable to formal support, such as support from health professionals and institutions (e.g., mental health intervention and treatment), among perinatal populations (Callister, Beckstrand & Corbett, 2011; Fonseca & Canavarro, 2017; Rice, Ingram, O’Mahen, 2022). Worthy of future empirical consideration is the role of grandparents in supporting parent-child wellbeing, particularly in the context of increasing rates of dually-employed parental and three-generation households in the United States due to multiple forces, including an aging population, the COVID-19 pandemic, increased financial stress, and the opioid epidemic (Dolbin-MacNab & O’Connell, 2021). Existing work highlights the promotive effects of grandparent support on perinatal mental health and subsequent child health and development (Riem et al., 2023; Sadruddin et al., 2019).

### 2.3. Youth socioemotional skill acquisition

#### 2.3.1. Supporting parent-child coregulation

Beginning during infancy, preventive interventions aimed at promoting responsive parent-child interactions during shared activities (e. g., parallel play, book reading) have been shown to have positive effects on early socioemotional skill development, including enhanced attention during play and reduced separation distress, hyperactivity, and externalizing behavior problems (Weisleder et al., 2016). Parents also help scaffold emerging self-regulatory abilities (e.g., emotion modulation) through modeling expressions of their own emotional experiences, labeling feelings (e.g., emotion talk), and intentional coaching of emotional issues (i.e., emotion socialization; Eisenberg et al. 1998; Silkenbeumer et al., 2016). Use of emotion talk (e.g., labeling feelings, drawing connections between feelings and their emotional expression) and emotion coaching (e.g., empathetic listening, validation, problem-solving skills) are of particular importance for preschool-aged children and increasing emotion talk can help disrupt links between family risk and poor child emotion regulation (Ellis et al., 2014), which is a transdiagnostic factor in the development of psychopathology (Aldao et al., 2016; Eisenberg et al., 2010).

#### 2.3.2. Supporting child self-regulation

Self-regulation, or the intrinsic processes that allow individuals to adapt to new contexts by adjusting mental and physiological states, is a transdiagnostic mechanism underlying psychopathology (Nigg, 2017) and a key target for universal prevention (Pandey et al., 2018; Traub & Boynton-Jarett, 2017). Executive function and effortful control are overlapping constructs that account for top-down aspects of self-regulation at the cognitive level (Nigg, 2017). Socioemotionally competent children must learn to modulate emotional and behavioral responses to engage in purposeful, goal-directed behavior, which involves executive functions (e.g., inhibitory control, working memory, and set-shifting) (Eisenberg & Zhou, 2016) as well as effortful control, a construct arising from temperament research that represents one’s ability to use cognitive control to influence emotion or behavior (Zhou, Chen & Main, 2012).

## 3. Universal prevention in practice

Relational health is a universal, biological imperative for children to be healthy and resilient (Humphreys et al., 2022). As such, targeting the key pillars of relational health, including screening for parent risk factors and resilience resources, promoting reciprocal social ties, and facilitating youth socioemotional regulation, warrants a *universal prevention* approach. Further, universal prevention offers unique population-level benefits relative to selective or indicated approaches (Fusar-Poli et al., 2021), as illustrated in the example of screening for postpartum depression. Although risk factors for postpartum depression are well-documented (Smorti et al., 2019), limiting screening to individuals who have an elevated risk profile based on these factors would nonetheless make up the minority of cases as lower-risk individuals comprise the majority of the population (i.e., the “prevention paradox”; Dodge & Goodman, 2019). In contrast, a universal prevention approach could have a larger impact on the population and has the potential to be more cost-effective in the long-term, as economies of scale can lower the per-family screening cost and costs of failing to prevent mental health problems far outpace those of service delivery (Dodge & Goodman, 2019; Johnson et al., 2023). In addition to the potential for greater impact and cost-effectiveness, when viewed as producing societal-level benefits rather than specialized services for a high-risk group, universal prevention efforts may advance equity (especially if providers rely on subjective judgment or biased instruments for selective/indicated prevention; Johnson et al., 2023), reduce stigma, and garner greater buy-in and sustained funding (Dodge & Goodman, 2019).

**Fig. 1.**
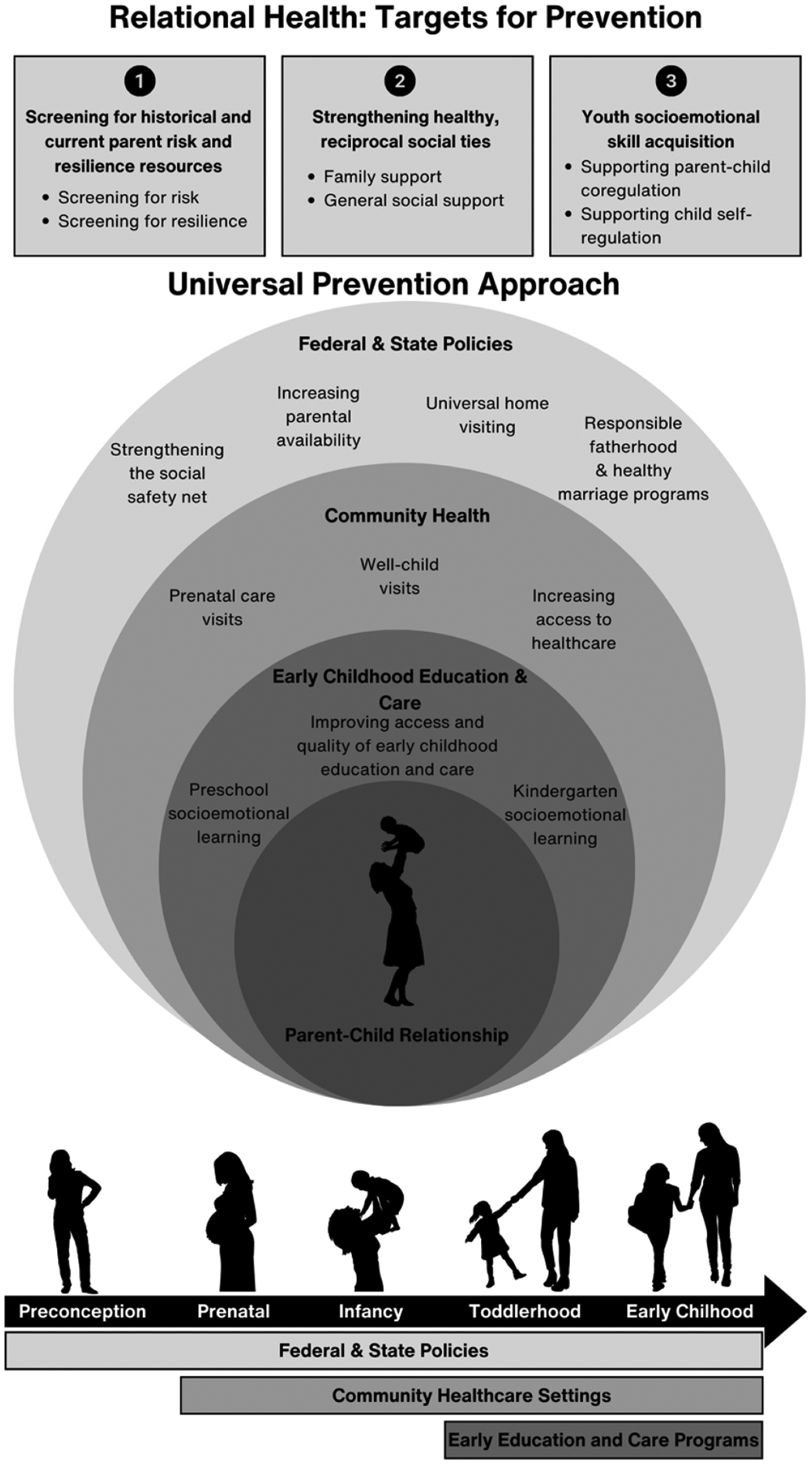
Note. We argue for a universal prevention approach, involving federal and state policies, community healthcare, and early education and care, that centers development and wellbeing in the context of the parent-child relationship. Through universal prevention strategies employed at these levels, we can identify risk factors and resilience resources for families, strengthen general and family social support for parents, and support emotion co-regulation and self-regulation, which have reciprocal and cascading benefits for both parent and child mental health. At the same time, we recognize that a universal prevention approach is necessary but not sufficient to prevent mental health problems for all children and families.

Although our approach to universal prevention is focused on promoting family resilience, we echo Humphreys et al. (2022) who argue that it is society that is ultimately responsible for supporting children’s and their families’ wellbeing. Through the lens of the bioecological model (Bronfenbrenner, 1979) and family resilience theory (Masten, 2018), the family microsystem is nested within a broader social ecology (Bronfenbrenner, 1979), whereby change at the individual and family levels is either constrained or facilitated by the broader sociocultural and political contexts in which they exist. As shown in Fig. 1, and described in the following sections, we offer actionable recommendations for universal prevention in the United States that can have profound impacts on relational health targets through changes to federal and state policies, community healthcare settings, and early childhood education and care programs.

### 3.1. Federal and state policies

#### 3.1.1. Strengthening the social safety net

Addressing economic hardship, which can lead to decreased caregiver availability, chaotic home environments, and harsh parenting styles, offers one avenue for supporting the parent-child interaction system and caregiver coregulation (Humphreys et al., 2022; Luby, Rogers & McLaughlin, 2022). We can be heartened that the calls of the American College of Obstetricians and Gynecologists, the American Medical Association and more than 275 other leading medical organizations for expanded postpartum coverage have been well-met, with nearly all states having adopted or being in the process of implementing postpartum Medicaid expansion (Health & Human Services, 2022) and can continue to encourage collective efforts to enact laws that support relational health. Other policies that are currently being evaluated in the U.S. context include mental health parity (i.e., equal coverage of mental and physical health conditions in insurance plans; So et al., 2019) and direct financial assistance to families, through cash transfers and expanded child care tax benefits (Luby et al., 2022).

#### 3.1.2. Increasing parental availability

Notably, despite meta-analytic evidence that maternity leave of at least 12 weeks is correlated with more positive mother-child interaction and better outcomes in maternal and child mental and physical health (Whitney et al., 2023), the United States is the only nation in the organization for economic co-operation and development (OECD) to have no national requirement for maternity leave. Legislative proposals for paid parental leave have the potential to reduce anticipatory and post-partum distress and increase caregiver availability and quality of regulation (Monk et al., 2019; Saxbe, Rossin-Slater & Goldenberg., 2018). For example, under the new Family and Medical Insurance Leave (FAMILY) Act in Congress (S.1714), workers would be able to take paid caregiving leave to support bonding with birth or adopted children and postpartum adjustment (which would support mothers and other primary caregivers), as well as other potential challenges to the family unit that may arise in other points in child development (e.g., deployment-related leave, safe leave in connection to sexual or domestic violence).

#### 3.1.3. Universal home visiting

Federal and state policies could support families during pregnancy through early childhood years by offering regular home visits to address families’ needs and strengths with a range of assessment resources, brief interventions to promote skill acquisition, and connection to social services (e.g., Dodge & Goodman, 2019). Home visiting has been shown to be beneficial in terms of both uncovering risk factors, such as maternal depression and violence exposure (Health Resources & Services Administration, 2024), and reducing child maltreatment (Avellar & Supplee, 2013). The health benefits of home visiting are enduring and also extend to future births (Holland et al., 2022). However, under current law, the maternal, infant, and early childhood home visiting (MIECHV) Program funds must be targeted to high-risk populations, such as low-income communities, pregnant women under 21 years of age, children with developmental delays, and families with a history of abuse, neglect, or substance use (Condon, 2019). Thus, we argue for universal provision of home visiting to new parents, as is done in several European countries (Condon, 2019). One such model is the Family Connect program, a universal postnatal nurse home-visiting program, which is designed to support all families in a community and has been shown to lead to improvements in positive parenting and reductions in postpartum depression at six months postpartum and longer-term positive impacts on child maltreatment (Baziyants et al., 2023).

#### 3.1.4. Responsible fatherhood and healthy marriage programs

In response to increasing numbers of children raised in one-parent households (US Census Bureau, 2017), federal and state governments have expanded funding for grant programs and activities that promote noncustodial parent involvement (often referred to as “responsible fatherhood initiatives”) through employment and training services, public campaigns that encourage noncustodial parents to become emotionally involved with their children, and counseling and relationship skills training. There are modest but significant benefits of fatherhood programs for co-parenting, parenting, and father involvement (Holmes et al., 2020). Though evaluation studies highlight challenges of serving low-income noncustodial parents (e.g., transportation, insufficient financial support, mental health concerns), programs that focus on co-parenting and the positive benefits of father involvement, raise awareness through trusted referral sources and in community settings commonly frequented by fathers, and explicitly welcome fathers to participate and involve them early on, are more likely to engage fathers and co-parenting couples (Lechowicz et al., 2019; Maxwell et al., 2012; Panter-Brick et al., 2014). Legislative support for Responsible Father-hood grants has also included expanded funding for Healthy Marriage Promotion grants programs, which must include activities to promote or sustain marriage (e.g., skills-based marriage education, dissemination of information on the causes of domestic violence and child abuse), to promote positive parenting (e.g., teaching positive parenting skills), and to promote economic stability. Meta-analytic evidence of all federally-funded programs supported through these grants similarly has demonstrated small but significant improvements in co-parenting, couple relationship quality, relationship skills, and parent mental health (Hawkins et al., 2022).

### 3.2. Community healthcare settings

#### 3.2.1. Prenatal care visits

Given that pregnant individuals regularly attend prenatal care visits, obstetricians are in an optimal position to identify families who may be at risk by screening mothers for ACES and perinatal mental health problems (Roubinov et al., 2021). Implementing early screening, prevention, and treatment of risk factors (particularly symptoms of depression and anxiety) as standard practice in prenatal care is now recommended (ACOG, 2018; Kilpatrick, Papile & Macones, 2017) and is found to be feasible and acceptable (Flanagan et al., 2018). Multi-level prevention and intervention that target co-parenting relational risk and resilience directly, such as screening for interpersonal violence (Chisholm, Bullock & Ferguson, 2017) or couples- or partner-based prenatal education and treatment (Suto et al., 2017), also have the potential to exert lasting impacts on parental and child wellbeing. In addition, the model of group prenatal care proposed by the American College of Obstetricians and Gynecologists (ACOG, 2018) has been shown to increase maternal perceived social support and in turn psychological wellbeing (Chae et al., 2017).

#### 3.2.2. Well-child visits

The American Academy of Pediatrics called for widespread screening of maternal postpartum depression during routine well-child visits (Earls, 2010) as an opportunity to identify mothers who are experiencing the effects of early childhood adversity and/or heightened perinatal distress (Roubinov et al., 2021). Similarly, the California Department of Healthcare Services (2020) recently launched the country’s first statewide effort to assess children for ACEs at routine pediatric primary care visits, and screening for parental ACEs may be included in future policy changes. Notably, empirical research demonstrates the feasibility of screening for parents’ histories of early adversity and perinatal depression at routine pediatric primary care visits (Earls, 2010; Earls & Hays, 2006; Gillepsie & Folger, 2017; Olson et al., 2006). Early identification and enhancement of PACEs during routine pediatric visits may be an effective prevention strategy to promote improved mental and physical health among children and families. In addition to screening for caregiver risk factors, providers can use the BCEs and/or PACEs assessment tools, reliable and valid brief questionnaires, during routine perinatal and/or pediatric primary care visits (Hays-Grudo & Morris, 2020; Merrick & Narayan, 2020). This dual emphasis on risk identification and resilience-promotion across generations provides a more comprehensive assessment of the family ecology. In contrast to a potentially stigmatizing focus on adversities, complementary assessment of family strengths (either historical or current resilience factors) can identify and enhance resilience, mitigate risks of screening with respect to service engagement, build trust, and enhance healthcare providers’ working relationships with families (Hays-Grudo et al., 2021; Leitch, 2017; Narayan et al., 2021). Evaluation of the implementation of combined risk and resilience screening efforts and their short- and long-term effects on referral rates, mental health and other specialist waitlists, receipt of services, mental health outcomes, and cost-effectiveness awaits future study, and should be considered within the local context as results may vary across healthcare settings (Barnett et al., 2021). For screening programs to be effective in promoting mental health and preventing future problems, they must both lead to the detection of family risks and resources and to the creation of personalized plans that support positive health and development and, if indicated, linkage to resources or mental health treatment (Barnett et al., 2021).

#### 3.2.3. Increasing access to healthcare

Several policy levers have been shown to increase access to mental health care, including location-based levers (e.g., integrated primary care and possibly telehealth/telemedicine) and insurance-based levers (e.g., increasing access to public health insurance or achieving mental health parity) (So et al., 2019). In addition to increasing access to mental health services, there must be appropriate guidance on handling positive screens and linkage to appropriate services. Although few pediatricians feel that diagnosis and management of maternal mental health symptoms would be within the scope of their practice, the majority report having delivered brief interventions (Olson et al., 2002). Linkages to mental health services can be facilitated by implementation of co-located and integrated treatment models, such as those that embed social work and/or mental health teams within primary care clinics (i.e., medical homes), and educational homes (i.e., that integrate multidisciplinary teams of school-based providers), and offer the opportunity to promote the caregiver-child dyad and may mitigate additional barriers for families already navigating contextual stressors that interfere with access to care (Earls, 2010; Hostutler et al., 2023; Ma et al., 2023).

### 3.3. Early childhood education and care programs

#### 3.3.1. Improving access and quality of early childhood education and care

Meta-analytic evidence convincingly demonstrates the importance of high-quality early childhood (0–5 years) education and care for reducing behavioral and socioemotional problems (von Suchodoletz et al., 2023); the benefits of high-quality care are robust and do not differ by children’s ethnicity or household socioeconomic resources (von Suchodoletz et al., 2023). Children who receive high-quality prekindergarten education through Head Start not only reap socioemotional and behavioral benefits, but also exhibit more positive parenting practices when raising their own children (Bauer & Schanzenbach, 2016), highlighting the intergenerational benefits of early childhood education. Though federal programs such as Head Start and Early Head Start can help families from lower-income backgrounds access high-quality early childhood education and care, such services are still out of reach for many families due to income eligibility requirements or residence in a “child care desert” (Malik et al., 2018). Just as limited financial support in the postpartum undermines parental availability, low wages for early educators threaten both the supply (e.g., due to high turnover rates, unfilled positions) and quality of care. Fortunately, there is widespread (≥ 90 %) support from the electorate (Halpin, Agne, & Omero, 2018) for supporting living wages for child care providers and recent Congressional action. By reissuing the Child Care and Development Block Grant program, through which states can subsidize child care costs and increase pay for early educators, Congress has expanded access to quality child care (Malik et al., 2018); yet, enhanced funding is needed to meet the nation’s current child care needs, without sacrificing quality of care, especially for care of infants and toddlers for whom families struggle most to find affordable and high-quality options (Schochet, 2018).

#### 3.3.2. Curriculum-based interventions: social and emotional learning

For children under 10 years of age, curriculum-based interventions in the school setting (e.g., social emotional learning [SEL]) are the most common and preferred intervention type to improve self-regulation (Pandey et al., 2018), though preventive programs that target parent emotion socialization skills also exist (England-Mason & Gonzalez, 2020). Beyond the teaching of academic content and skills, substantial meta-analytic research supports the need for schools to provide both universal instruction in SEL, such that children gain competencies in self-awareness, self-management, social awareness, relationship skills, and responsible decision making (CASEL, 2020). SEL has repeatedly been shown to promote long-term success and have positive benefits for behavioral, social, emotional, and academic outcomes (Blewitt et al., 2018; Corcoran et al., 2018; Mahoney et al., 2018; Murano, Sawyer & Lipnevich, 2020). Though the content may differ to align with developmental appropriateness, the effectiveness of socioemotional (SEL) interventions is similar for preschoolers and K-12 students (Murano et al., 2020). The benefit of SEL programming is maximized when routinely and systematically implemented in preschool and continued through high school (Berman et al., 2018; Dusenbury & Weissberg, 2018).

##### 3.3.2.1. Preschool and kindergarten sel interventions.

During the preschool years, evidence-based SEL interventions should focus on teaching skills needed to prepare for the transition to formal schooling. As preschoolers’ capacity for metacognitive thinking is often still emerging (Dimmitt & McCormick, 2012), curriculum at this age should focus on social and communication skills and has been shown to reduce behavior problems, particularly among at-risk students (Murano et al., 2020) who perhaps received less early scaffolding through parent-child interactions. Programs should seek to incorporate movement, creativity, and social relations into SEL curriculum to support the rapid development in executive functions occurring during early childhood (Bierman & Motamedi, 2015; Diamond & Ling, 2016). During the transition to kindergarten, children experience a concurrent increase in demands for attentional, emotional, and behavioral control as their classrooms become more structured and academic learning goals become more apparent.

##### 3.3.2.2. Promoting competence in sel curricula among early childhood educators and benefits of parental involvement.

Preschoolers remain heavily reliant on external supports (e.g., parents, teachers) and continuous access to high-quality early childhood education supports evolving interpersonal and school readiness skills (Archambault, Côté & Raynault, 2020; Tran, Luchters & Fisher, 2017). Through involvement of parents and teachers, the opportunities for embedding direct instruction and daily practice of specific skills into everyday activities grow exponentially and, in turn, contributes to the effectiveness of the interventions (McClelland et al., 2017). Though level of parent involvement can vary program to program, there is general support that their involvement and home-based activities add value to effectiveness of interventions to improve socioemotional skills (Smith et al., 2020).

In early education settings, early childhood educators play arguably the most vital role in effective implementation of SEL-based curricula. As such, they should be provided with professional development supports and specific curriculum about SEL interventions, from its empirical basis through program delivery, and this information should be provided in a digestible format given the range of educational qualifications obtained by early childhood educators (Gomez, Kagan & Fox, 2019). Specific SEL-focused training (e.g., multiday training sessions) serves to improve teacher effectiveness in implementation of interventions, supports their own building of SEL-related skills (McClelland et al., 2017), and can buffer against teacher burnout which, in turn, improves the quality of teacher-child interactions (Sandilos, Goble & Schwartz, 2020). Further, teachers would benefit from increased access to reimbursable consultations with mental health specialists to support an individual child’s mental health needs (Burak & Rolfes-Haase, 2018).

## 4. When universal prevention is not enough

Among certain children and families facing heightened risk due to individual or structural factors, such as systemic racism and discrimination and additional barriers to accessing child care and healthcare due to geographic location, socioeconomic status, universal prevention may not be enough, warranting a blend of universal prevention with additional targeted prevention and intervention efforts (Fusar-Poli et al., 2021). Such a tiered universal and targeted approach is evident in best practice recommendations for medically-hospitalized infants and their caregivers. Parent-infant separation throughout infant hospitalization, intensive medical care, and infant medical complexity place caregivers of infants hospitalized in neonatal intensive care and cardiovascular intensive care units at elevated risk for postpartum mood and anxiety disorders and medical traumatic stress (Grunberg et al., 2022), with potential impacts on the developing caregiver-infant dyadic relationship (Kasparian et al., 2019; Tesson et al., 2023). Hospitalized infants face additional contextual adversity, including exposure to the noises and lights of an intensive care environment, isolation or reduced contact with caregivers, and numerous medical procedures (Givrad, Hartzell & Scala., 2021). In these medical units, universal parental mental health screening and infant developmental risk screening is the recommended best practice (Hynan et al., 2015; Kasparian et al., 2019). In combination with universal screening, additional targeted psychosocial follow up and dyadic intervention is recommended among parents and infants who screen at-risk for poor psychosocial or neurodevelopmental outcomes (e. g., due to elevated symptoms on mental health screeners or infant medical complexity; Hynan et al., 2015; Givrad et al., 2021). A similar tiered approach of universal screening with additional targeted prevention and intervention can also facilitate addressing positive perinatal screens for depression and other common perinatal mental health problems in perinatal and pediatric primary care (e.g., as part of systems-enhanced care; Gjerdingen & Yawn, 2007). Meta-analytic work suggests universal (redesigned postnatal care) and targeted approaches (e.g., interpersonal therapy) can be integrated to improve postpartum depression and anxiety (Fusar-Poli et al., 2021); downstream benefits for offspring outcomes may also be enhanced through two-generation interventions, such as programs that integrate depression treatment with evidence-based parenting supports (Roubinov et al., 2023).

## 5. Conclusion

Consistent with extensive developmental theory on the bidirectionality of the parent-child relationship (Paschall & Mastergeorge, 2016), distinct lines of research converge on the notion that parent and child wellbeing is inextricably linked. Secure parent-child attachment and parent-child coregulation provides the foundation for children’s emerging biobehavioral regulatory capacity; yet, parent risk factors, including psychopathology, can compromise these early relational origins of child psychological functioning. At the same time, youth temperamental negativity and childhood behavior problems can elicit poor parenting, compromise dyadic processes, and exacerbate parent mental health problems. Attending to the interdependence between children’s and their parents’ mental health during sensitive windows for the developing relationship and for child mental health can thus have transformative impacts for both members of the parent-child dyad. Yet, recognizing the relational context of child development does not mean that influences on child development are limited to this context; rather it is our hope that increased awareness of the reciprocal and interdependent influences between parent and child will lead stakeholders to advocate for expanded funding and changes at federal, state, and community levels to promote family resilience. We have offered specific recommendations for government policies, community healthcare settings, and early childhood education and care that could be enacted in the United States to help screen for parent risk and resilience factors, strengthen social ties, and promote youth socioemotional acquisition – taken together, meaningful change in these areas has the potential to strengthen the parent-child dyad and prevent mental health problems across their lifetimes and into future generations.

## Figures and Tables

**Table 1:** Empirical Support for Relational Health Prevention Targets and Strategies that Affect these Targets.

	Key reviews	Evidence of effective strategies		
Prevention targets	Outcomes	Federal & state policies	Community healthcare settings	Early education and care programs
Screening for parental risk and resilience^1^	Maternal depression (O’Connor et al., 2023); General parental mental health ( Merrick & Narayan, 2020); *Possible Indirect Effects via:* Increased referral to services ( Barnett e al., 2021; Loveday et al., 2022)	Mother-infant and early childhood home visiting (MIECHV) program ( Avellar & Supplee, 2013); *Possible Indirect Effects via:* Paid maternity/family leave (Van Niel et al., 2020; Lichtman-Sadot & Bell, 2017); Increased access to public health insurance and mental health parity (So et al., 2019)	Prenatal care appointments ( Kilpatrick et al., 2017); postpartum OBGYN visits (Tully et al., 2017); Well-child visits (Kia-Keating et al., 2019; Selvaraj et al., 2019); HealthySteps pediatric primary care program (Briggs et al., 2016); Integrated primary care (So et al., 2019)	
Strengthening healthy, reciprocal social ties	Maternal depression (Ni & Siew Lien, 2024); Maternal depression, anxiety, and self-harm (Bedaso et al., 2021); General maternal mental health (Atzl et al., 2019; Razurel et al., 2013); Child externalizing behavior problems ( Sarkadi et al., 2008); *Possible Indirect Effects via* Maternal health care use (Atzl et al., 2019); Maternal sensitive parenting (Ni & Siew Lien, 2024); Parental co-regulation (Paley & Hajal, 2022); Mother-child relationship quality (Atzl et al., 2019)	Longer (≥12 weeks) and paid maternal maternity leave (Van Niel et al., 2020; Whitney et al., 2023); Responsible Fatherhood programs ( Holmes et al., 2020); Couple relationship education (Hawkins et al., 2022)	Group based prenatal care (Chae et al., 2017); Internet support and local models of postpartum home visiting (Sharifipour et al., 2022)	
Youth Socioemotional Skill Aquisition	Lifespan internalizing and externalizing problems (Aldao, 2016; Diamond, 2016); Youth internalizing and externalizing problems (Eisenberg et al., 2010)	Paid maternity leave (Van Niel et al., 2020); child care and development fund (CCDF) grant program (von Suchodoletz et al., 2023): Early Head Start and Head Start Programs (Bauer & Schanzenbach, 2016; Love et al., 2005); Couple relationship education (Hawkins et al., 2022); MIECHV (Avellar & Supplee, 2013)	Pediatric practitioners (Traub & Boynton-Jarrett, 2017)	PATHS and Incredible Years programs in Head Start (Sandilos, Goble & Schwartz, 2020); Universal public preschool socio-emotional learning curriculum (SEL; Bierman et al., 2023; Mondi, Giovanelli, & Reynolds, 2021); Parent involvement in school-based SEL (McClelland et al., 2017; Smith et al., 2020); Parent emotion socialization programs ( England-Mason & Gonzalez, 2020)

*Note*. Recommendations are informed by conceptual and action theories of mediation (MacKinnon et al., 2002). Key prevention targets are derived from conceptual theories, based on their associations with parent and child mental health outcomes (as documented in key narrative, systematic, or meta-analytic reviews of the empirical literature). Effective strategies are derived from action theories, based on the demonstrated ability of these strategies to affect change in the prevention targets.

1 To date, reviews of the mental health benefits of screening have focused on screening for parental risk and not on screening for parental resilience factors.

## Footnotes

2 Though our recommendations apply to parents broadly defined, we acknowledge that the majority of the extant theoretical and empirical evidence of bidirectionality has focused on mother-child dyads (e.g., Paschall & Mastergeorge, 2015).
